# Artificial Intelligence–Powered Electrocardiogram Detecting Culprit Vessel Blood Flow Abnormality: AI-ECG TIMI Study Design and Rationale

**DOI:** 10.1016/j.jscai.2024.102494

**Published:** 2025-01-13

**Authors:** Robert Herman, Timea Kisova, Marta Belmonte, Adriaan Wilgenhof, Gabor Toth, Anthony Demolder, Adam Rafajdus, H. Pendell Meyers, Stephen W. Smith, Jozef Bartunek, Emanuele Barbato

**Affiliations:** a Department of Advanced Biomedical Sciences, University of Naples Federico II, Naples, Italy; b Cardiovascular Centre Aalst, Aalst, Belgium; c Powerful Medical, Bratislava, Slovakia; d Department of Clinical and Molecular Medicine, Faculty of Medicine and Psychology, Sapienza University of Rome, Italy; e Division of Cardiology, Medical University of Graz, Graz, Austria; f Department of Emergency Medicine, Carolinas Medical Center, Charlotte, North Carolina; g Department of Emergency Medicine, University of Minnesota, Minneapolis, Minnesota; h Department of Emergency Medicine, Hennepin Healthcare, Minneapolis, Minnesota

**Keywords:** acute coronary syndrome, artificial intelligence, coronary pathophysiology, electrocardiogram, non–ST-elevation myocardial infarction, ST-elevation myocardial infarction

## Abstract

**Background:**

The 12-lead electrocardiogram (ECG) is the gold standard for detecting patients who will benefit from emergent revascularization due to occlusive myocardial infarction (OMI). However, the pathophysiology of acute coronary syndromes (ACS) is dynamic, and nearly half of patients with OMI do not present with typical ST elevation or have dynamic ECG changes due to spontaneous recanalization before invasive coronary angiography (ICA). Recently, an ECG-based artificial intelligence (AI) model was developed using expert interpretation of OMI. However, its performance is limited to retrospective evaluation of ECGs recorded minutes to hours before ICA.

**Methods:**

The AI-ECG thrombolysis in myocardial infarction (TIMI) study is an investigator-initiated prospective multicenter registry planning to enroll over 700 consecutive patients with ACS undergoing ICA in 9 centers across Europe. For all participants, a standard 10-second 12-lead ECG will be recorded at the time of coronary angiography. The primary end point is the AI model’s ability to identify patients with an actively occluded (TIMI 0-1) culprit coronary artery at the time of invasive coronary angiography using only single-standard 12-lead ECGs. Standardized angiograms will be used as a reference standard.

**Conclusions:**

AI-ECG TIMI is the first prospective registry of consecutive patients with ACS with standard 12-lead ECGs recorded at the very moment of ICA. This study will help characterize ECG findings of abnormal myocardial perfusion due to acute active ischemia and prospectively validate an AI model’s ability to detect them.

The current classification of acute coronary syndrome (ACS) identifies patients who will benefit from emergent reperfusion therapy based on electrocardiographic (ECG) ST-segment elevation (ST elevation myocardial infarction [STEMI]).^1^ However, the majority of patients with ACS present without typical ST-elevation, and at least 25% of these patients have an acutely occluded culprit coronary artery (occlusion myocardial infarction [OMI]) at the time of invasive coronary angiography (ICA).^2^, ^3^, ^4^

The new 2023 ESC Clinical Practice Guidelines for Management of ACS, suggest a plethora of STEMI-equivalent ECG criteria (eg, de Winter, hyperacute T-waves, posterior STEMI, or modified Sgarbossa criteria for left bundle branch block).^5^ However, their recognition at the first point of patient contact is suboptimal, leading to delayed invasive management, which is associated with 2-fold higher short-term and long-term mortality.^2^^,^^3^^,^^6^ Growing evidence calls for a new paradigm, going beyond the individual features of the ECG, categorizing patients as either presenting acute OMI or non-OMI.^3^^,^^4^^,^^7^, ^8^, ^9^ Moreover, the pathophysiology of ACS due to thrombotic occlusive coronary stenosis is often dynamic and impacts the ECG appearance throughout the index hospitalization. One-third of occluded culprit lesions in patients with true STEMI recanalize before ICA.^10^^,^^11^ Thus, based on their ECG findings before ICA, patients with OMI could further be subclassified into actively occluded (STEMI equivalents) or partially reperfused (eg, Wellens syndrome).

Recently, an artificial intelligence (AI) model was developed using a cohort of 18,616 patients with suspected ACS and with ECG expert annotations and angiographic outcomes, demonstrating 2-fold higher sensitivity compared with standard ECG assessment for STEMI criteria in detecting OMI in a multicenter international validation cohort.^12^ Since the validation was retrospective with variable times to coronary angiography, the study could not confirm whether the patients had acute active coronary occlusion (culprit thrombolysis in myocardial infarction [TIMI] 0-1) at the time of the ECG recording. Moreover, the AI model detected a broad spectrum of patients with OMI without explicitly differentiating between patients with active occlusion at the time of ECG recording and those with spontaneous recanalization after recent active occlusion.

A new AI model in development, AI-ECG TIMI, aims to subclassify acute OMI based on the state of the culprit coronary artery at the time of recording the ECG into (1) actively occluded (TIMI 0-1) and (2) open/reperfused (TIMI 2-3). AI-ECG TIMI is developed using ECGs of patients with ACS and OMI adjudicated by expert ECG interpretation in the context of outcome data available.

This prospective multicenter registry will enroll more than 700 consecutive patients with ACS referred for ICA as clinically indicated. In the AI-ECG TIMI study, standard 12-lead ECGs recorded shortly before ICA will be correlated with culprit coronary vessel blood flow assessed by the TIMI grade flow. In this study, we aim to (1) validate a new version of the AI model differentiating between persistent coronary occlusion and reperfused culprit coronary artery; (2) correlate definitions of OMI based on routine angiographic and laboratory parameters to abnormal myocardial perfusion; (3) describe the prevalence of active OMI without typical ST elevation (STEMI equivalent) ECG patterns during known, angiographically proven, persistent occlusion.

## Materials and methods

### Study design

The AI-ECG TIMI study is an investigator-initiated prospective, multicenter registry of ACS patients undergoing ICA as clinically indicated. The study is registered at ClinicalTrials.gov (NCT06528821). A total of 709 patients with ACS will be enrolled at 9 investigational sites in Belgium, Italy, and Austria, with each site responsible for an equal enrolment target of around 80 patients (Central Illustration). An academic core laboratory will analyze electrocardiographic and angiographic data. End points will be evaluated by independent statistical analysis using prespecified contingency tables.

**Central Illustration:**
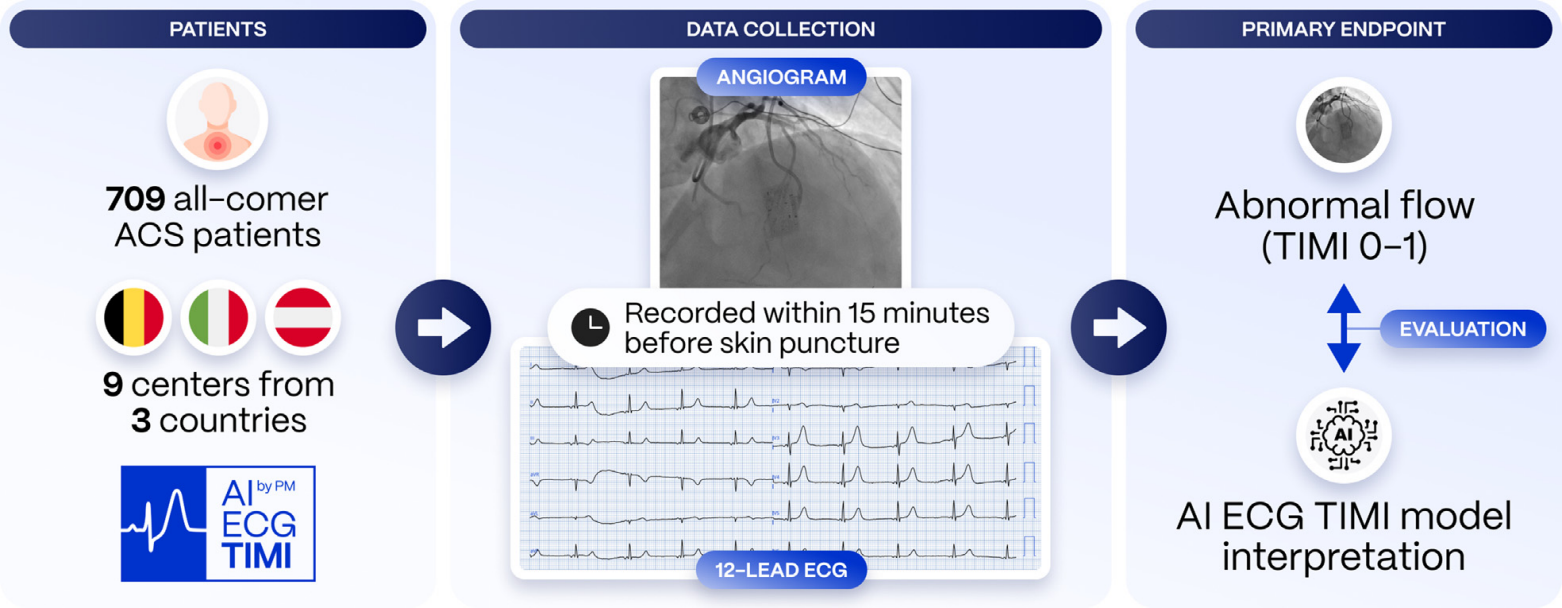
The AI-ECG TIMI study is a multicenter prospective registry of patients with ACS undergoing standard 10-second 12-lead ECG monitoring shortly before invasive coronary angiography. This database will be used to evaluate an AI-powered ECG model detecting ACS assessed by abnormal culprit coronary blood flow (TIMI 0-1). ACS, acute coronary syndromes; AI, artificial intelligence; ECG, electrocardiogram; TIMI, thrombolysis in myocardial infarction.

### Screening and eligibility criteria

Adult patients presenting to the emergency department triage (walk-in, referral, or via emergency medical services) with a clinical suspicion of ACS undergoing 12-lead ECG screening will be considered for initial eligibility assessment. Consecutive patients with a working diagnosis of ACS (both STEMI and non-STEMI) admitted for ICA as per standard practice will be included in the study. Interventional cardiologists will enroll patients before the ICA procedure, ensuring all eligibility criteria are met. A full list of inclusion and exclusion criteria is available in Table 1.

**Table 1:** Inclusion and exclusion criteria.

Inclusion criteria

Potential subjects must meet all of the following criteria to be eligible for inclusion in the study: • Age ≥ 18 y • Patients with acute coronary syndromes undergoing invasive coronary angiography as clinically indicated • Availability of a standard 12-lead electrocardiogram performed at invasive coronary angiogram before percutaneous coronary intervention (maximum within 15 min before vascular access)
Exclusion criteria

Potential subjects will be excluded if any of the following conditions apply: • Individuals presenting for a nonemergent (elective) cardiac catheterization • Individuals presenting with chronic coronary syndrome or stable angina symptoms • Individuals without symptoms suspicious for acute coronary syndromes • Individuals with contraindications for cardiac catheterization • Individuals participating in clinical investigations or trials that may interfere with the procedures or outcomes of this study

### Index procedures

For all included patients with ACS undergoing ICA, 10-second 12-lead ECGs with standard lead placement will be acquired at 3 standardized time points: T1, before ICA (within 15 minutes before skin puncture); T2, after termination of standard diagnostic angiography or percutaneous coronary interventions (PCIs); T3, 90 minutes after PCI. Diagnostic angiography will be performed based on standardized projections—left anterior descending coronary artery: right anterior oblique (RAO) 30° − cranial (CRA) 30° projection; left circumflex coronary artery: RAO 40° − caudal 30° projection; right coronary artery: left anterior oblique 30° − CRA 15° or RAO 30° − CRA 0° projections. Angiograms will be recorded at a minimum of 25 frames per second and for a duration sufficient to capture the filling of the venous coronary system and backflow of the contrast agent into the aorta. For all patients undergoing PCI, as indicated, an additional standardized angiogram of the culprit vessel(s) will be acquired after PCI. For patients without a culprit vessel requiring urgent revascularization, T2 and T3 time points will be skipped.

### Core laboratory analysis

Quantitative analysis of coronary angiograms will be performed by an independent core laboratory at the Sapienza University in Rome, Italy. QAngio XA 3D/QFR Software (Medis Medical Imaging) will be used to assess minimum lumen area, percent area stenosis, reference vessel areas, lesion length, TIMI frame count, TIMI myocardial perfusion grading myocardial blush grade, and quantitative flow ratio.^13^ The baseline SYNTAX score, the residual SYNTAX score, and the Δ SYNTAX score will be calculated.

### Study outcomes

The primary end point is the AI-ECG TIMI model’s ability to identify patients with an actively occluded (TIMI 0-1) culprit coronary artery using only single-standard 12-lead ECG recorded at the time of ICA (T1). The primary end point will assess the accuracy, sensitivity, specificity, positive and negative predictive values, and F1 score of the AI model. Secondary end points will include the evaluation of the AI-ECG TIMI model performance in subgroups, benchmark analysis, and patient-related end points (Table 2).

**Table 2:** Secondary end points.

**AI-ECG TIMI model end points**
1. AI-ECG TIMI model performance across subgroups: (1) demographic, (2) risk factors and comorbidities, (c) electrocardiographic, and (4) culprit artery territory 2. Correlation of AI-ECG TIMI model’s raw numeric predictions (between 0 and 1) with myocardial perfusion grading after percutaneous coronary interventions
**Benchmark analysis end points**
1. Validation of expert ECG interpretation blinded to all clinical information: a. Accuracy, sensitivity, specificity, positive and negative predictive values of expert interpretation of active OMI, reperfused OMI, and entirety of OMI b. Interrater variability of expert interpretation of active OMI, reperfused OMI, and entirety of OMI. 2. Benchmark of machine-based ECG interpretation vs blinded expert ECG interpretation vs OMI AI-ECG model to detect OMI on pre-ICA ECGs
**Angiographic definition end points**
1. OMI definitions using routine angiographic and laboratory parameters will be correlated to abnormal myocardial perfusion using correlation coefficients: a. OMI defined as acute culprit with (1) TIMI <3; OR (2) TIMI 3 with vessel stenosis of >70% b. OMI defined as acute culprit with (1) TIMI <3; OR (2) TIMI 3 and very high peak troponin elevation (hs-cTnT ≥ 1000 ng/L, cTnI of > 10.0 ng/mL, or cTnT of > 1.0 ng/mL or hs-cTnI > 5000 ng/L) c. OMI defined as acute culprit with TIMI <3 d. OMI defined as acute culprit with TIMI 0-1 2. Prevalence of STEMI-equivalent ECG patterns and their correlation to TIMI flow grade at angiography
**Patient-related end points**
1. Percentage (%) of AI ruled-in patients (OMI AI-ECG Model–positive interpretation within 10 min of recording ECG) at admission invasively managed within 2 h stratified by presence of formal STEMI criteria 2. D2B times of AI ruled-in OMI without ST-elevation criteria (NSTEMI-OMI) with ECG scanned into PMcardio within 5 min of recording the initial ECG 3. Percentage (%) of OMI patients stratified by presence of formal STEMI millimeter criteria at admission 4. OMI patients stratified based on ECG expert-rated OMI state (active vs reperfused) at presentation and culprit coronary flow at angiography 5. NSTEMI-OMI stratified according to immediate (<2 h) vs SoC (>2 h) invasive management (median): (1) peak troponin (maximum rise before fall), (2) left ventricular ejection fraction at discharge, (3) length of hospital stay, (4) length of cardiac care unit or intensive care unit stay, and (5) presence of life-threatening arrhythmias

AI, artificial intelligence; cTnI, cardiac troponin I; cTnT, cTnT, cardiac troponin T test; ECG, electrocardiogram; hs, high-sensitivity; ICA, invasive coronary angiography; NSTEMI, non–ST-elevation myocardial infarction; OMI, occlusive myocardial infarction; STEMI, ST-elevation myocardial infarction; SoC, standard of care; TIMI, thrombolysis in myocardial infarction.

### Statistical analysis

#### Sample size and interim analysis

The study sample size was estimated based on ECG expert performance in detecting active OMI in 466 ECGs of patients with ACS undergoing ICA within 2 hours. The experts could detect active occlusion with 91.2% sensitivity and 53% specificity. However, the analysis likely underestimated specificity, as 52% (53 of 101) of the expert false positives were patients with true STEMI with a patent vessel (TIMI 3 flow) at coronary angiography. The prevalence of active occlusion (TIMI 0-1 flow) at coronary angiography in an all-comer ACS population undergoing ICA is estimated to be around 40%.

Drawing from the aforementioned assumptions, we estimate that a cohort of 709 consecutive patients with ACS would be sufficient to accurately evaluate an AI model detecting active coronary occlusion with a precision of 5% and a 95% 2-sided CI, assuming a dropout rate of 10%. Due to the probable underestimation of ECG expert specificity, an interim analysis will be performed after 200 successfully enrolled patients. In the interim analysis, ECG experts will review the presence of active coronary occlusion on all ECGs recorded at the time of coronary angiography for all successfully enrolled patients. The sensitivity and specificity of ECG experts detecting TIMI 0-1 will be recalculated, and the required sample size will be updated.

#### Primary and secondary end points

A contingency table will be created to assess the primary end point comparing the diagnostic performance of the AI-ECG TIMI on the 12-lead ECG recorded at ICA compared with the reference standard of angiographically measured abnormal coronary blood flow. For all evaluation metrics, 95% CIs will be estimated by 10,000 iterations of the bootstrap method.^14^ A *P* value of <.05 will be considered statistically significant. For secondary end points, forest plots will evaluate demographic, electrocardiographic, and culprit artery subgroup model performance. For each subgroup, *P* values will compare performance within a subgroup to the performance of the model on the overall patient population. Some subgroups may be combined depending on data availability.

### Data collection

Study-related data will be entered using an electronic data capture system (Castor EDC; Castor). Admission ECGs will be scanned in using the CE-certified PMcardio smartphone application (Powerful Medical). All investigators will follow Good Clinical Practice, relevant laws and regulations, and the study-related protocol.

## Discussion

The AI-ECG TIMI study is the first prospective, multicenter registry of consecutive patients with undergoing standard 12-lead ECG at the time of ICA. The study is designed to capture findings of acute ischemia on ECGs collected throughout the dynamic continuum of ACS due to thrombotic plaque rupture at 3 standardized time points. This database will serve as an accurate reference standard to evaluate a new AI-ECG TIMI model differentiating between active and reperfused OMI.

The application of AI in detecting cardiovascular diseases is advancing rapidly, with the availability of large digital data sets positioning ECG-based AI research at the forefront.^12^^,^^15^, ^16^, ^17^, ^18^, ^19^ However, a significant limitation of existing ECG data sets is their retrospective nature and reliance on subjective human interpretation, which introduces variability and limits the precision needed for clinical reliability.^20^ McCabe et al^21^ showed that the interrater variability for ECG interpretation of STEMI among physicians was 0.33, reflecting poor agreement.^21^ Additionally, deep neural networks may identify patterns in data that are undetectable by human interpreters. To unlock the full potential of AI-driven ECG models and achieve favorable performance warranting clinical adoption, these models must be calibrated and validated against objective, outcome-based data. In the context of ACS detection, this requires validation against angiographic end points to ensure accuracy and clinical relevance.

Previous studies have investigated ECG findings of active coronary occlusion or high-grade culprit stenosis.^22^ However, in all of these studies, the ECG and angiograms were obtained at separate time points, typically 2 hours apart. This temporal gap complicates the evaluation of ECG findings and their correlation to culprit vessel blood flow and limits the adoption of a new paradigm in the management of ACS, focusing on the state of the coronary artery rather than millimeter criteria of ST elevation.^9^ One-third of patients with OMI and typical ST-elevation millimeter criteria on the index ECG have an open artery before PCI, thus the vessel status at ICA underestimates the true number of patients presenting with actively occluded culprit vessels.^11^

The STAFF III database continuously recorded 12-lead ECGs in 104 patients undergoing balloon inflation during PCI.^23^ However, this database only included patients undergoing elective balloon PCI, and artificial balloon occlusion might not be reflective of acute coronary occlusion owing to thrombotic plaque rupture.

In the AI-ECG TIMI Study, 12-lead ECGs will be acquired in all-comer patients with ACS at 3 systematic time points (index, immediately before and after ICA, and upon conclusion of mechanical reperfusion defined as 90 minutes after angioplasty). Standardized angiograms will be acquired to evaluate myocardial perfusion using quantified myocardial blush grading. The data collected in this study will be used to validate an AI model detecting active occlusion.

Several limitations of this study design merit considerations. First, the study will only include patients with ACSs undergoing ICA. Second, this study is not powered for clinical end points such as infarct size or mortality and patient follow-up is not performed. The primary aim is to gather a high-quality, prospective cohort of consecutive patients with ACS with independently adjudicated angiographic outcomes to validate and calibrate the AI-ECG model accurately. Moreover, while the study promotes consecutive enrollment, certain high-risk patients, such as those with hemodynamic instability, may be excluded if a 12-lead ECG cannot be recorded within minutes before vascular access.

The AI-ECG TIMI study will contribute to the growing evidence that calls for a new paradigm in the management of ACS focusing on the state of the culprit coronary artery. These efforts will help characterize ECG findings of abnormal myocardial perfusion due to acute active ischemia and provide insights into the prevalence of previously reported ECG manifestations.
